# Nutritional Composition, Amino Acid, Fatty Acid Profiles and Total Polyphenols of Sweet Apricot Kernels

**DOI:** 10.1155/ijfo/4555989

**Published:** 2026-08-22

**Authors:** Jamila Smanalieva, Janyl Iskakova, Nurzat Shaikieva, Anke Foerster, Anne Hellwig, Thomas Henle

**Affiliations:** ^1^ Department of Food Chemistry, Faculty of Chemistry and Food Chemistry, Technische Universität Dresden, Dresden, Germany, tu-dresden.de; ^2^ Department of Food Science and Technology, Institute of Technology, Kyrgyz State Technical University After I. Razzakov, Bishkek, Kyrgyzstan, kstu.kg; ^3^ Environmental Engineering Department, Engineering Faculty, Kyrgyz-Turkish Manas University, Bishkek, Kyrgyzstan, manas.edu.kg; ^4^ Chemical Engineering Department, Engineering Faculty, Kyrgyz-Turkish Manas University, Bishkek, Kyrgyzstan, manas.edu.kg

**Keywords:** amino acids, apricots, carbohydrates, fatty acids, kernel, minerals, oil, protein

## Abstract

Sweet apricot kernels (*Prunus armeniaca* L.) are a by‐product of apricot processing; however, they remain underutilised. This study investigated the nutritional composition and bioactive properties of sweet apricot kernels from three Central Asian cultivars: Suhany, Kandek and Kurmayi. Apricot kernels are a rich source of nutrients and bioactive compounds, highlighting their potential as functional food ingredients. The kernels contained 3.3%–4.7% moisture, 40.0%–49.9% fat, 17.9%–18.9% protein, 8.1%–14.7% crude fibre and 2.2%–2.7% ash. Glutamic acid was the predominant amino acid, accounting for 24%–25% of the total amino acid content. The kernels were rich in unsaturated fatty acids, with oleic acid (C18:1, *cis*‐9) representing 24%–29% and linoleic acid (C18:2, *cis*‐9,12) representing 5%–9% of total fat content. Essential minerals, including zinc, magnesium and copper, were present at nutritionally relevant levels, with a 30 g serving providing up to 11% of the daily requirement for zinc, 7%–20% for magnesium and 78% for copper. Total polyphenol content ranged from 29 to 194.8 mg GAE/100 g dry weight, whereas vitamin C content ranged from 2.2 to 7.1 mg/100 g. The amygdalin content in sweet apricot kernels is generally low (~0.06–0.88 g/100 g) compared to bitter kernels and is safe for consumption. These findings provide valuable data for updating food‐composition databases.

## 1. Introduction

Apricots are widely cultivated in Mediterranean and Central Asian countries and contribute significantly to regional economies [1]. Apricot production in Central Asia exceeds 0.6 million tonnes annually, accounting for approximately 16% of global production [2]. Apricot kernels, which are enclosed within the hard shell of the fruit stone, represent valuable by‐products of apricot processing. They are recognised as a source of protein, essential fatty acids, minerals and dietary fibre [3, 4]. Apricot kernels contain up to 50% fat, 25% protein and minor amounts of carbohydrates (2.8%); vitamins, including tocopherols; and dietary fibre [5–8]. The lipid fraction is dominated by unsaturated fatty acids, primarily oleic acid (31%–67%) and linoleic acid (20%–43%). In addition, the kernels are rich in tocopherols, niacin, magnesium (Mg) and potassium (K), which contribute to metabolic regulation and mineral homeostasis [9]. Previous studies have shown that consumption of apricot kernels improves biochemical parameters in rats with hepatic fibrosis, potentially due to their high levels of oleic acid and polyphenols.

Apricot kernels are classified as sweet or bitter. Bitter kernels contain significantly higher concentrations of the cyanogenic glycoside amygdalin (~2.7–6.3 g/100 g), whereas sweet kernels contain substantially lower amounts (~0.06–0.88 g/100 g) [10]. This difference in amygdalin concentration determines the characteristic taste of the kernels and explains the higher risk of cyanide toxicity associated with bitter kernels. Although amygdalin itself is nontoxic, enzymatic hydrolysis by *β*‐glucosidase during chewing, crushing or soaking releases hydrogen cyanide (HCN), a toxic compound [11]. Bitter kernels are commonly used for oil extraction in the cosmetic and pharmaceutical industries due to their antioxidant and antimicrobial properties [7, 12, 13]. In contrast, roasted sweet kernels are consumed as snacks and widely incorporated into bakery and confectionery products. Persipan, produced from apricot kernels and sugar, serves as an alternative to marzipan [7].

Apricots are commonly treated with sulphur dioxide (SO_2_) or sulphite compounds before sun‐drying to preserve colour, protect nutrients and extend shelf life. Typically, sulphuring is performed by burning ~1.5–2 g of sulphur per kilogramme of fresh fruit for 6–12 h [1]. This treatment helps preserve *β*‐carotene, organic acids and vitamin C during drying and storage [6, 7]. However, excessive sulphite residues may raise health concerns and contribute to environmental pollution. In addition, growing demand for organic products has stimulated interest in SO_2_‐free preservation alternatives. The effect of sulphuring on the nutritional value of apricot kernels has not yet been studied.

Food composition databases provide essential nutritional information that supports public health policies and the development of dietary guidelines aimed at preventing noncommunicable diseases [14]. A review of major food composition databases, including Food Data Central (United States), the Bundeslebensmittelschlüssel (Germany), FoodExplorer (EuroFIR) and the Russian Food Composition Tables and Database, showed that apricot kernel oil is included in most databases. However, information on the nutritional composition of apricot kernels themselves remains limited. Among the databases reviewed, only EuroFIR (Estonia) provides compositional data for apricot kernels (macronutrients and some minerals). Furthermore, the amino acid profile was not reported, and information on fatty acid composition is incomplete in the available records. These gaps highlight the need for comprehensive nutritional characterisation of apricot kernels and their inclusion in national and international food composition databases. Therefore, this study was aimed at characterising the nutritional composition of apricot kernels from three apricot cultivars, including protein, fat, sugars, fibre, vitamin C, polyphenols and essential minerals. The data obtained were compared with the published literature and existing food composition databases, thereby contributing to the refinement and updating of these resources.

## 2. Materials and Methods

Dried apricot kernels were obtained from local farmers in Batken, Kyrgyz Republic, in March 2024. The apricot cultivars used in this study and their corresponding abbreviations are presented in Table 1. Kernel samples from both sulphured and unsulphured fruits of the Kurmayi cultivar were additionally analysed as a case study to evaluate the effects of SO_2_ treatment on the nutritional value of apricot kernels. For each cultivar, 250 g of fruit stones was manually cracked, and the edible kernels were separated from the shells, weighed, ground into a fine powder and thoroughly homogenised. The resulting kernel powder was used for all subsequent analyses. Before analysis, the samples were stored at −20°C.

**Table 1:** Samples′ varieties, country of origin and abbreviations.

Cultivar and sample name	Treatment and drying technology	Country of origin
Suhany	Sun‐dried kernel, unsulphured	Batken, Kyrgyzstan
Suhany, oil	Pressed oil from sun‐dried, unsulphured kernels	Batken, Kyrgyzstan
Kandek	Sun‐dried kernel, unsulphured	Batken, Kyrgyzstan
Kurmaiy	Sun‐dried kernel, unsulphured	Batken, Kyrgyzstan
Kurmaiy, sulphured	Sun‐dried kernel, sulphured	Batken, Kyrgyzstan

### 2.1. Analyses of Macronutrients

Moisture content was determined by heating 5.0 ± 0.5 g of ground, air‐dried apricot kernel samples in a drying oven at 105°C until constant weight was achieved. Nitrogen content was determined using the improved Kjeldahl method (AOAC 950.48) with a Büchi Labortechnik AG analytical system (Flawil, Switzerland) and converted to crude protein using a nitrogen‐to‐protein conversion factor of 5.30 [15].

Crude fat content was determined using the Soxhlet extraction method (AOAC 2003.05), with petroleum ether as the extraction solvent [15].

Crude fibre content was determined by first defatting 2 g of the ground sample with petroleum ether, followed by digestion with 1.25% (*v*/*v*) H_2_SO_4_ and 1.25% (*v*/*v*) NaOH for 30 min. The digested material was filtered through a cotton filter, and the residue was ashed at 550°C for 4–6 h, cooled in a desiccator and weighed (AOAC 962.09) [15].

Ash content was determined according to the AOAC method 942.05 [15]. All analyses were performed in triplicate.

### 2.2. Determination of Glucose, Fructose and Sucrose Contents

Glucose, fructose, sucrose and sorbitol contents were determined by extracting approximately 10–13 mg of each ground sample with 10 mL of ultrapure water in an ultrasonic bath for 15 min. The extracts were filtered through Whatman No. 4 filter paper and subsequently diluted 20‐fold before analysis. Carbohydrates were quantified using high‐performance anion‐exchange chromatography with pulsed amperometric detection (HPAEC‐PAD) on a Dionex AS‐AP system (Thermo Scientific, Waltham, MA, United States), following the method described by Barber et al. [16]. Separation was performed on a Dionex CarboPac PA1 column (2 × 250 mm). The injection volume was 10 *μ*L. Isocratic elution was carried out using 200 mM NaOH as the mobile phase at a flow rate of 0.25 mL/min. Calibration curves were prepared using external standards of glucose, fructose and sucrose at concentrations ranging from 1 to 200 *μ*mol/L. Data acquisition and chromatographic analysis were performed using Chromeleon 7 software.

### 2.3. Determination of Ascorbic Acid Content

Ascorbic acid content was determined by reversed‐phase HPLC with UV detection, according to the method described by [17]. Ground sample (1.250 g) was mixed with 15 mL of 1.5% metaphosphoric acid, shaken for 15 min and sonicated for 3 min. The extract was then diluted to a final volume of 25 mL with the same solution (1.5% metaphosphoric acid). The resulting solution was filtered through Whatman No. 4 filter paper, then through a 0.45 *μ*m membrane filter (RC), before chromatographic analysis.

Analysis was performed using an Agilent 1100 HPLC system (Agilent Technologies, United States). A stock solution of vitamin C (1 g/L) was prepared using 1.5% metaphosphoric acid, and calibration standards were prepared in the concentration range of 50–1000 *μ*L/mL. The calibration equation was *y* = 16.183*x* − 20.348, with a coefficient of determination (*R* ^2^) of 0.9974.

The mobile phase was prepared by dissolving 1.361 g of KH_2_PO_4_ in 600 mL of bidistilled water, followed by the addition of 200 mg of tetrabutylammonium hydrogen phosphate. The pH was adjusted to 2.5 using dilute H_3_PO_4_ solution, and the final volume was adjusted to 1000 mL with distilled water. The mobile phase was filtered through a 0.45 *μ*m membrane filter (RC 55) and degassed in an ultrasonic bath for 5 min. Chromatographic separation was performed on a ProntoSIL 60‐5 Phenyl column (250 × 4.6 mm; Knauer, Wissenschaftliche Geräte). The flow rate was set at 0.8 mL/min, and detection was carried out using a diode‐array detector at 243 nm, with the column temperature maintained at 40°C. Each sample was analysed in duplicate.

### 2.4. Determination of Antioxidant Activity

Antioxidant activity was evaluated using the 2,2‐diphenyl‐1‐picrylhydrazyl (DPPH) radical‐scavenging assay according to the method described by Hangun‐Balkir and McKenney [18]. A 0.01% (*w*/*w*) DPPH solution in 80% ethanol was prepared as the free‐radical reagent. For extraction, 1 g of ground apricot kernel was mixed with 20 mL of 80% ethanol and stirred for 20 min. The mixture was then centrifuged at 5000 rpm for 3 min. The supernatant was collected and diluted to obtain five concentrations (1, 2.5, 5, 7.5, 10, 15 and 20 mg/mL) using 80% ethanol.

For the antioxidant assay, 2 mL of each extract solution was mixed with 2 mL of a 0.01% DPPH solution prepared in 80% ethanol. The mixtures were thoroughly shaken and incubated at room temperature for 30 min. The control solution consisted of 2 mL of 80% ethanol and 2 mL of DPPH solution. The absorbance of both the control and sample solutions was measured at 517 nm using a UV‐Vis spectrophotometer (Tecan Infinite 200Pro, Germany). The antioxidant activity was calculated according to Equation (1):

(1) %AA=Abs control−Abs sampleAbscontrol·100

where *%*AA represents the antioxidant activity (per cent inhibition), Abs sample is the absorbance of the sample and Abs control is the absorbance of the control. The results were expressed as IC_50_ values, defined as the concentration of extract required to inhibit 50% of DPPH free radicals. IC_50_ values were determined from inhibition curves obtained by plotting percentage inhibition against extract concentration. All measurements were performed in triplicate.

### 2.5. Determination of Total Phenolic Content (TPC)

The TPC was determined using the Folin–Ciocalteu method according to the procedure described by [19]. A 10% methanolic extract of the kernels was prepared, homogenised and filtered through Whatman No. 2 filter paper. A 20‐*μ*L aliquot of the extract was mixed with 100 *μ*L of Folin–Ciocalteu reagent (Merck, Darmstadt, Germany) and allowed to react for 5 min. Subsequently, 300 *μ*L of sodium carbonate solution (Na_2_CO_3_, 20 g/L, Merck KGaA, Darmstadt, Germany) was added, followed by 1580 *μ*L of distilled water to obtain a final volume of 2 mL. The reaction mixture was incubated in the dark at room temperature for 30 min. The absorbance was then measured at 765 nm against a reagent blank using a UV‐Vis spectrophotometer (Tecan Infinite 200Pro, Germany).

A calibration curve was prepared using gallic acid standard solutions in the concentration range of 0.5–5 mg/mL. The calibration equation was *y* = 0.496*x* with a coefficient of determination (*R* ^2^) of 0.9899. TPC was expressed as milligrammes of gallic acid equivalents per 100 g sample. All analyses were performed in triplicate.

### 2.6. Determination of Fatty Acid Composition of Kernels

Fatty acid composition was determined by converting triglycerides into fatty acid methyl esters (FAMEs) through alkaline transesterification using potassium methanolate (5% KOH in methanol). The resulting FAMEs were extracted with cyclohexane and dilute sulphuric acid and subsequently analysed by gas chromatography coupled with a flame ionisation detector (GC‐FID; Agilent Technologies 7820A GC System, United States). Separation was performed using a ZB‐FFAP capillary column (30 m × 0.25 mm i.d., 0.25‐*μ*m film thickness) according to the method described in Ulambayar et al. [20].

Identification of individual fatty acids was carried out by comparing retention times with those of a certified FAME standard mixture (Supelco 37 Component FAME Mix, Sigma‐Aldrich, United States). In addition to apricot kernel samples, cold‐pressed apricot kernel oil obtained from the Suhany cultivar was analysed to assess the effect of the oil extraction (pressing) process on fatty acid composition. All analyses were performed in triplicate.

### 2.7. Determination of the Amino Acid Composition of the Kernel

The amino acid composition of apricot kernels was determined by ion‐exchange chromatography with postcolumn ninhydrin derivatisation using a Sykam S 433 Amino Acid Analyser (Chromatographie Vertriebs GmbH, Fürstenfeldbruck, Germany). Briefly, 0.2 g of the sample was hydrolysed with 10 mL of 6 M HCl in a sealed long‐neck ampoule and incubated at 110°C for 23 h. Following hydrolysis, a 1.5 mL aliquot of the hydrolysate was evaporated to dryness. The residue was reconstituted in 2 mL of lithium citrate buffer (0.12 M, pH 2.2) and filtered through a regenerated cellulose membrane filter (0.45‐*μ*m pore size).

Chromatographic separation was performed on a cation‐exchange column (LCA K07/Li, 7 *μ*m, 4.6 × 150 mm) maintained at 42°C. The flow rates were set at 16 mL/h for the buffer solutions and 10 mL/h for the ninhydrin solution. Four buffer systems were used for amino acid separation: A‐1 (0.12 M lithium citrate, pH 2.9), B‐1 (0.3 M lithium citrate, pH 4.2), C‐4 (0.3 M lithium citrate, pH 8.0) and a regeneration buffer (0.5 M lithium hydroxide).

Identification and quantification of amino acids were carried out using a standard amino acid mixture obtained from Serva Electrophoresis GmbH (Heidelberg, Germany). In this method, glutamic acid was determined as the sum of glutamic acid and glutamine, whereas aspartic acid was determined as the sum of aspartic acid and asparagine. Tryptophan could not be quantified because it is degraded during acid hydrolysis. Likewise, cystine and methionine were not determined due to oxidative losses occurring during sample preparation. Detection was performed at 440 nm for proline and at 570 nm for all other amino acids [21]. Each sample was analysed in duplicate.

### 2.8. Statistical Analysis

All results were expressed as mean ± standard deviation (SD) based on three independent replicates (*n* = 3) reported per 100 g of sample. Statistical differences among the three apricot kernel varieties were evaluated using one‐way analysis of variance (ANOVA) followed by Duncan′s multiple range test as a post hoc comparison. Statistical analyses were performed using IBM SPSS Statistics Version 20 (IBM Corp., Armonk, NY, United States). Differences were considered statistically significant at *p* < 0.05.

## 3. Results and Discussion

### 3.1. Macronutrient Composition of Apricot Kernels

The moisture content of the apricot kernels ranged from 3.3 to 4.7 g/100 g, whereas ash content varied between 2.2 and 2.7 g/100 g (Table 2). These moisture values were comparable to or slightly lower than those reported in previous studies. For instance, apricot kernels from Malatya, Türkiye, contained 3.4% moisture and 2.5% ash [4], whereas kernels from New Zealand contained 4.7% moisture and 2.9% ash [22]. The analysed kernels contained 40.0%–49.9% fat and 17.9%–18.9% protein. These values are within the ranges reported in the literature, where protein content varies from 14.6% to 28.8% [3, 23–27] and fat content from 40.8% to 57.1 g/100 g [24, 27]. Compared with New Zealand apricot kernels, which contain 52% fat and 20.6% protein [22], the cultivars examined in the present study showed slightly lower values. Such variation is attributable to differences in genotype, growing conditions, climate and geographic origin.

**Table 2:** Macronutrient composition of apricot kernels.

Samples	Moisture (g/100 g)	Fat (g/100 g)	Protein (g/100 g)	Glucose (g/100 g)	Fructose (g/100 g)	Crude fibre (g/100 g)	Ash (g/100 g)	NFE
Suhany	3.30 ± 0.09^b^	44.00 ± 0.04^b^	17.87 ± 2.46^a^	0.53 ± 0.18^b^	0.70 ± 0.16^c^	8.80 ± 0.60^c^	2.73 ± 0.06^a^	22.06^a^
Kandek	4.66 ± 0.15^a^	48.00 ± 1.37^a^	18.89 ± 0.64^a^	0.68 ± 0.02^b^	2.82 ± 0.37^b^	8.12 ± 2.56^c^	2.22 ± 0.06^a^	16.65^b^
Kurmaiy	3.75 ± 0.07^b^	49.93 ± 0.41^a^	18.70 ± 1.07^a^	3.60 ± 0.07^a^	3.27 ± 1.94^a^	10.32 ± 2.25^b^	2.69 ± 0.24^a^	8.31^c^
Kurmaiy, sulphured	3.33 ± 0.21^b^	44.63 ± 8.69^b^	17.87 ± 1.59^a^	3.65 ± 0.09^a^	3.32 ± 0.04^a^	14.66 ± 0.92^a^	2.69 ± 0.25^a^	7.36^с^
EuroFIR [23]	8	50.70	25.00	0.3		0.8	2.11	

*Note:* Data are represented as mean ± standard deviation. Different superscript letters within a column indicate significant differences (ANOVA, multiple comparison: Duncan′s method) (*p* < 0.05). Crude fibre refers to plant polysaccharides and lignin that are not digested by enzymes in the human digestive tract.

Abbreviation: NFE, nitrogen‐free extract, *%* = 100 − (moisture*%* + crude protein*%* + crude fat*%* + crude fibre*%* + ash*%*).

Only low concentrations of glucose and fructose were detected, whereas sucrose and sorbitol were not present in the analysed kernels. Crude fibre content ranged from 8.1 to 14.7 g/100 g, exceeding most previously reported values for apricot kernels (0.8–4.3 g/100 g) [23, 24], but remaining comparable to those reported for sweet apricot kernels (12.5 g/100 g) [25] and almonds (10.2–15.2 g/100 g) [23]. It should also be noted that crude fibre values are not directly comparable with total dietary fibre because the crude fibre method quantifies mainly insoluble fibre fractions, including cellulose, lignin and pentosans, while excluding most soluble fibre components, such as inulin and pectin. Therefore, the higher crude fibre values observed in this study are unlikely to be explained by methodological differences between crude fibre and total dietary fibre determinations. Instead, the discrepancy may be due to cultivar characteristics, sample preparation procedures and analytical approaches. For example, in the study of [24], the seed coat was removed before analysis, which may have significantly reduced the measured fibre content. Overall, significant differences in macronutrient composition were observed among apricot cultivars, suggesting a strong genetic influence on kernel composition. In contrast, sulphur treatment did not appear to affect the nutritional composition of the kernels.

### 3.2. Amino Acid Composition of Apricot Kernels

The results of the amino acid analysis of apricot kernels are presented in Table 3. Apricot kernels contained 17 amino acids, among which glutamic acid was the most abundant, accounting for 18.5%–26% of the total amino acid content. In the present study, glutamic acid represents the combined content of glutamic acid and glutamine. This amino acid has also been reported as the predominant amino acid in almond, peach and mango kernels [28, 29]. Siberian apricot kernels, a variety of apricot, were reported to contain 29 g/100 g of protein and to be rich in glutamic acid (26.5%), aspartic acid (11.3%) and arginine (10.1%) [30]. Although glutamic acid is a nonessential amino acid, it plays a crucial role in numerous biological processes, including metabolism, neurotransmission and plant growth. Owing to its functional properties, it is also widely used in the food and agricultural industries [31].

**Table 3:** Amino acid profile of apricot kernels (g/100 g).

Amino acid	Suhany		Kandek		Kurmayi	
AA	Mean	SD	Mean	SD	Mean	SD
Asn + Asp	1.34	0.25	0.98	0.08	1.14	0.12
Thr^a^	0.37	0.07	0.30	0.02	0.39	0.08
Ser	0.54	0.10	0.41	0.03	0.49	0.09
Gln + Glu	3.05	0.60	2.18	0.17	2.54	0.28
Gly	0.65	0.13	0.49	0.04	0.60	0.06
Ala	0.59	0.11	0.46	0.04	0.48	0.05
Cys	0.00	0.00	0.00	0.00	0.07	0.00
Val^a^	0.51	0.09	0.40	0.03	0.53	0.08
Met^a^	0.00	0.00	0.00	0.00	0.12	0.17
Ile^a^	0.45	0.08	0.34	0.03	0.37	0.04
Leu^a^	0.84	0.15	0.65	0.05	0.71	0.09
Tyr	0.38	0.07	0.29	0.02	0.18	0.01
Phe^a^	0.65	0.12	0.50	0.03	0.78	0.15
His^a^	0.29	0.06	0.22	0.02	0.05	0.07
Lys^a^	0.39	0.08	0.31	0.03	0.22	0.02
Arg	1.20	0.24	0.86	0.07	0.97	0.10
Pro	0.55	0.10	0.41	0.04	0.58	0.04
Total AA	11.8	2.25	8.8	0.7	10.22	1.45
SUM EAA	3.5	0.65	2.72	0.21	3.17	0.7

*Note:* Data are represented as means (*n* = 3). No significant differences were observed between groups for all data in the same item (*p* > 0.05).

Abbreviations: AA, amino acid; Ala, alanine; Arg, arginine; Asp + Asn, aspartic acid + asparagine; Cys, cysteine; EAA, essential amino acids; Gln + Glu, sum of glutamine and glutamic acid; Gly, glycine; His, histidine; Ile, isoleucine; Leu, leucine; Lys, lysine; Met, methionine; Phe, phenylalanine; Pro, proline; SD, standard deviation; Ser, serine; Thr, threonine; Trp, tryptophan; Tyr, tyrosine; Val, valine.

^a^Essential amino acids.

In contrast to our findings, apricot kernels from India were reported to contain high levels of aspartic acid and tyrosine, which together accounted for more than 40% of the total amino acid content [8]. The next most abundant amino acids were arginine and leucine, with concentrations ranging from 0.97 to 1.20 mg/100 g kernels and from 0.70 to 0.88 mg/100 g of kernels, respectively. No significant differences were observed in the amino acid composition among the analysed samples.

The apricot kernels studied contained 29.7%–31.1% essential amino acids relative to total amino acid content, with leucine, phenylalanine and valine being the predominant essential amino acids. In almonds (*Prunus dulcis*), essential amino acids account for 36%–45% of the total amino acid content, with leucine, phenylalanine and isoleucine being the major amino acids [28]. Therefore, apricot kernels are comparable to almonds in terms of amino acid quality. Moreover, these essential amino acids play important physiological roles. Leucine and valine are branched‐chain amino acids involved in muscle protein synthesis, energy metabolism and tissue repair, whereas phenylalanine serves as a precursor to tyrosine and several neurotransmitters, including dopamine, epinephrine and norepinephrine, which support normal nervous system function [32]. The presence of these essential amino acids highlights the potential nutritional and functional value of apricot kernels as a source of dietary protein.

It should be noted that the near‐zero concentrations of methionine and cysteine observed in the present study are likely attributable to methodological limitations rather than their actual absence. Sulphur‐containing amino acids are susceptible to oxidation and degradation during acid hydrolysis, which may result in their underestimation or nondetection. More accurate quantification can be achieved by oxidising methionine and cysteine before hydrolysis and subsequently measuring their stable oxidation products after hydrolysis. However, this approach requires a modified procedure and a different separation method.

### 3.3. Fatty Acid Composition of the Apricot Kernel and Oil

Table 4 presents the fatty acid profiles of apricot kernels and their oil. The major fatty acids in the apricot kernel oil were oleic acid (C18:1, *cis*‐9), accounting for 64% of total fatty acids, followed by linoleic acid (C18:2, *cis*‐9,12; 19.25%) and palmitic acid (C16:0; 3.76%). Smaller amounts of stearic, linolenic and palmitoleic acids were also detected. Similar results were reported for apricot kernel oil from Poland, where oleic acid was the predominant fatty acid (60.01%–70.56%), followed by linoleic acid (19.74%–23.52%), palmitic acid (2.35%–5.97%), stearic acid (0.8%–1.5%) and palmitoleic acid (0.2%–0.9%) [33].

**Table 4:** The fatty acid profile of apricot kernels and oil (g/100 g).

Fatty acids	Kandek		Kurmaiy		Suhany		Suhany, kern oil	
	Mean	SD	Mean	SD	Mean	SD	Mean	SD
Caproic acid (C6:0)	nd		0.014^a^	0.00	nd		nd	
Caprylic acid (C8:0)	0.06	0.01	0.07	0.02	0.06	0.01	0.06	0.01
Myristic acid (C14:0)	nd		nd		nd		0.02^a^	0.00
Palmitic acid (C16:0)	1.86^b^	0.01	1.37^c^	0.11	1.89^b^	0.01	3.76^a^	0.06
Palmitoleic acid (C16:1, *cis*‐9)	0.30^b^	0.00	0.21^c^	0.02	0.34^b^	0.00	0.56^a^	0.01
Heptadecenoic (С17:1, *cis*‐10)	0.04^c^	0.00	0.03^c^	0.00	0.05^b^	0.00	0.11^a^	0.00
Stearic acid (C18:0)	0.40^b^	0.04	0.37^b^	0.01	0.48^a^	0.03	nd	
Oleic acid (C18:1, *cis*‐9)	29.83^b^	0.40	24.60^c^	2.30	29.06^b^	0.27	64.37^a^	0.70
Linoleic acid (C18:2, *cis*‐9,12)	8.23	0.10	5.86	0.53	9.01	0.02	19.25	0.15
*γ*‐Linolenic acid (C18:3, *cis*‐6,9,12)	nd		0.01	0.01	nd		0.01	0.01
*α*‐Linolenic acid (C18:3, *cis*‐9,12,15)	0.03	0.01	0.03	0.01	0.03	0.01	0.09	0.01
Arachidic acid (C20:0)	0.04	0.03	0.04	0.00	0.05	0.01	0.10	0.01
Erucic acid (22:1, *cis*‐13)	0.06	0.01	0.05	0.01	0.05	0.01	0.11	0.00
Behenic acid (C22:0)	nd		nd		nd		0.02	0.02
Docosadienoic acid (C22:2. c13.c16)	0.07^b^	0.02	0.15	0.05	0.08^b^	0.01	0.19^a^	0.05
Tricosylic acid (C23:0)	nd		0.02	0.03	nd		0.02	0.02
Lignoceric acid (24:0)	0.02	0.01	0.01	0.01	0.02	0.01	0.04	0.01
Docosahexaenoic acid (22:6)	0.02^d^	0.01	0.05^a^	0.01	0.01	0.00	0.04^b^	0.01
Sum of identified FA	44.84	0.20	36.51	0.25	44.79	0.01	92.29	0.35
Unknown	0.77	0.191	1.49	0.25	0.80	0.01	2.70	0.35
SFA	2.21		1.61		2.29		4.39	
MUFA	30.22		24.89		29.51		65.16	
PUFA	8.330		6.07		9.12		19.53	
Sum of all FA	46.40 ^b^		38.00 ^c^		45.60 ^b^		95.00 ^a^	

*Note:* Data are represented as means (*n* = 3). Different superscript letters within a column indicate significant differences (ANOVA, multiple comparison: Duncan′s method) (*p* ≤ 0.05).

Abbreviation: SD, standard deviation.

One hundred grammes of the edible portion of apricot kernels contained 24–29 g of oleic acid (C18:1, *cis*‐9), 5–9 g of linoleic acid (C18:2, *cis*‐9,12) and 1.3–1.8 g of palmitic acid (C16:0). Alajil et al. [8] reported oleic acid contents ranging from 10.73 to 29.79 g/100 g, linoleic acid contents from 4.68 to 17.69 g/100 g and palmitic acid contents from 0.31 to 1.09 g/100 g in apricot kernels. These findings suggest that the fatty acid composition of apricot kernels may be influenced by cultivar and geographical origin. Linoleic acid (C18:2, *cis*‐9,12) is an essential omega‐6 fatty acid that contributes to the maintenance of normal blood cholesterol levels and cardiovascular health. Oleic acid has been associated with beneficial effects on lipid metabolism, and replacing saturated fatty acids with oleic acid contributes to the maintenance of normal LDL‐cholesterol levels [32]. According to the National Research Council (United States), the adequate intake of linoleic acid is approximately 8–9 g/day (about 4% of total energy intake in a 2000 kcal diet) [34]. Based on the fatty acid composition determined in the present study, consumption of 30 g of sweet apricot kernels would provide approximately 7.2–8.7 g oleic acid. This corresponds to approximately 27%–34% of the recommended daily intake of linoleic acid and represents a substantial dietary source of oleic acid.

### 3.4. Mineral Composition of Apricot Kernels

Table 5 presents the macro‐ and micronutrient contents of apricot kernels. The K and Mg contents of apricot kernels were comparable to those reported for almonds, which contain approximately 835 and 170 mg/100 g, respectively [23, 32]. Among the studied cultivars, the highest K concentration was in the Suhany cultivar (812.79 mg/100 g). The average K content of the investigated samples was 564.36 mg/100 g, which is consistent with values reported in the German Food Composition Database (Version 3.2) for apricot kernels [23]. K is an essential electrolyte involved in maintaining fluid balance, supporting muscle function and regulating heart rhythm [35]. Considering that a typical serving size of nuts is approximately 30 g, apricot kernels can provide about 2.5%–7% of the recommended daily K intake (RDI) [36].

**Table 5:** Mineral elements of apricot kernels (mg/100 g).

Samples	Zn	Fe	Mn	Cu	Mg	Ca	Na	K
Suhany	3.22^b^	1.90^a^	0.64^a^	0.84^a^	156.13^b^	105.09^b^	0.75^a^	812.79^a^
Kandek	4.00^a^	2.20^a^	0.96^a^	1.03^a^	216.13^a^	166.18^a^	1.69^a^	589.35^b^
Kurmaiy	2.05^b^	0.93^a^	0.42^a^	0.44^a^	91.85^c^	79.76^c^	1.10^a^	290.95^c^
Apricot kern [23]	4.25	9.2	0.3	1.026	234	26	4	599

*Note:* Data are represented as means (*n* = 3), standard deviation ≤ 0.01. Different superscript letters within a column indicate significant differences (ANOVA, multiple comparison: Duncan′s method) (*p* ≤ 0.05).

The highest Mg concentration was found in the Kandek cultivar. Mg is an essential mineral involved in numerous physiological processes, including DNA synthesis, protein synthesis, energy production, blood glucose regulation, transmembrane ion transport, oxidative phosphorylation and glycolysis, and normal muscle and nervous system function [34]. The RDI for Mg is approximately 350 mg for adults [36]. Therefore, 30 g of apricot kernels can provide 7%–20% of the daily Mg requirement.

The calcium (Ca) concentration in the investigated apricot kernels ranged from 79.8 to 166.2 mg/100 g, exceeding values reported in previous studies [23]. Ca is essential for the development and maintenance of bones and teeth, muscle contraction and vitamin D–related metabolic processes [37]. Given the RDI of 1000 mg for adults [36], a 30 g serving of apricot kernels can provide approximately 2.4%–5.0% of the daily Ca requirement.

Copper (Cu) concentrations ranged from 0.44 mg/100 g in the Kurmayi cultivar to 1.3 mg/100 g in the Kandek cultivar. Cu plays an important role in haemoglobin synthesis, iron (Fe) metabolism, energy production, enzymatic reactions and biological electron transfer processes [38]. The RDI of Cu is approximately 0.5–1 mg for children and 2 mg for adults [36]. Accordingly, a 30 g serving of apricot kernels can provide approximately 13%–78% of the daily Cu requirement, depending on cultivar and age group.

Manganese (Mn) concentrations ranged from 0.42 to 0.96 mg/100 g. Mn functions as an indirect antioxidant, contributes to bone and connective tissue formation and serves as a cofactor for enzymes involved in amino acid, carbohydrate and catecholamine metabolism. It is also required for cholesterol and nucleotide synthesis [39]. Based on the recommended daily intake of 5 mg/day for adults [36], a 30 g serving of apricot kernels can provide approximately 2.4%–5.4% of the daily Mn requirement.

Apricot kernels contained 2–4 mg zinc (Zn) per 100 g, which is consistent with values reported for apricot kernels from Turkey (1.18–4.24 mg/100 g) [40]. Zn is involved in numerous metabolic processes and acts as a structural or catalytic component of many enzymes. It is essential for the metabolism of carbohydrates, proteins, fats and nucleic acids and plays an important role in gene expression, hormone regulation and vitamin metabolism [38, 41]. With an RDI of approximately 8–11 mg/day for adults [36], 30 g of apricot kernels can provide about 8%–11% of the daily Zn requirement.

The Fe concentration was within the range previously reported in the literature (1.07–7.49 mg/100 g) [40, 42], although the German Food Composition Database reports a higher value of 9.5 mg/100 g [23]. These differences may be attributed to variations in cultivar, geographical origin, environmental conditions or analytical methodology. Among the studied cultivars, Kandek exhibited the highest Fe concentration. Fe is an essential component of haemoglobin, myoglobin and numerous enzymes involved in oxygen transport and cellular metabolism [43]. The RDI of Fe is approximately 10 mg/day for men and 18 mg/day for women [34]. Therefore, a 30 g serving of apricot kernels can provide approximately 3%–13% of the daily Fe requirement, depending on cultivar.

### 3.5. Bioactive Components of Apricot Kernels

Table 6 presents the total polyphenol content, ascorbic acid content and antioxidant activity of the studied apricot kernels. The TPC of the investigated samples ranged from 29 to 194.8 mg GAE/100 g dry weight (DW), which is consistent with previously reported values. Alajil et al. [8] reported TPC values ranging from 39.7 to 130.9 mg GAE/100 g DW in apricot kernels, whereas Korekar et al. [44] reported values between 92.2 and 162.1 mg GAE/100 g DW. Considerably higher concentrations of phenolic compounds have been reported in apricot kernel peels (874.5 mg GAE/100 g), exceeding those found in peels of almonds (*Amygdalus pedunculata* Pall.), which contained 781.8 mg GAE/100 g [45].

**Table 6:** Bioactive components of apricot kernels.

Samples	TPC (mg GAE/100 g DW)	Vitamin C content (mg/100 g)	Antioxidant capacity (IC_50_) (mg/mL)
Suhany	29.5 ± 9.4^c^	2.2 ± 1.7^b^	26.5 ± 1.7^c^
Kandek	38.3 ± 0.3^b^	2.8 ± 0.3^b^	14.7 ± 1.5^b^
Kurmaiy	40.5 ± 0.5^b^	2.3 ± 0.3^b^	15.0 ± 0.5^c^
Kurmay, sulphured	194.8 ± 1.3^a^	7.1 ± 0.2^a^	12.5 ± 1.7^a^

*Note:* Data are represented as means (*n* = 3) ± standard deviation. Different superscript letters within a column indicate significant differences (*p* < 0.05) (ANOVA, multiple comparison: Duncan′s method).

Abbreviations: DW, dry weight; TPC, total polyphenol content.

The antioxidant activity of the samples was evaluated using the DPPH radical‐scavenging assay and expressed as the inhibitory concentration (IC_50_). Previous studies reported IC_50_ values ranging from 43.8 to 123.4 mg/mL for apricot kernels [44]. The IC_50_ values obtained in the present study were generally lower, indicating stronger radical‐scavenging activity. However, the results of the in vitro DPPH assay should be interpreted with caution, as they cannot be directly extrapolated into in vivo biological effects. The DPPH assay primarily reflects the chemical ability of compounds to neutralise free radicals under controlled laboratory conditions and is therefore most suitable for preliminary screening of antioxidant potential. Confirmation of biological relevance requires further investigation using appropriate in vivo or cellular models.

The ascorbic acid content of the investigated apricot kernels ranged from 2.2 to 7.1 mg/100 g. A literature review notes that apricot kernels are rich in ascorbic acid but give no numeric vitamin C concentration for kernels. Sulphured kernels exhibited significantly higher levels of total phenolics and ascorbic acid than untreated samples. In addition, the lowest IC_50_ values were observed in sulphured kernels, indicating greater antioxidant activity. The enhanced antioxidant capacity may be related to higher ascorbic acid content and improved preservation of phenolic compounds during processing. Nevertheless, the comparison between sulphured and nonsulphured samples may have been influenced by other factors, including cultivar differences, processing conditions and storage history. Therefore, the observed differences cannot be attributed exclusively to SO_2_ treatment, which should be considered a limitation of the present study.

### 3.6. Amygdalin and Safety Considerations

A limitation of the present study is that amygdalin, the principal cyanogenic glycoside in apricot kernels, was not directly quantified in the analysed samples. Therefore, safety considerations related to cyanogenic potential were assessed based on published data for sweet apricot cultivars. Previous studies have demonstrated that sweet apricot kernels generally contain substantially lower amygdalin concentrations (32–235 *μ*g/g DW) than bitter genotypes (538–24,000 *μ*g/g DW), although considerable variation has been reported depending on cultivar, geographical origin and analytical methodology [46]. Similar concentrations, ranging from trace levels to approximately 235 *μ*g/g DW, have also been reported for commercially available sweet apricots [46]. Assuming a conservative worst‐case scenario based on the highest reported concentration (235 *μ*g/g DW), consumption of a 30 g serving of apricot kernels would provide approximately 7.05 mg of amygdalin. Given that 1 g of amygdalin can theoretically release approximately 59 mg of HCN, this amount would correspond to a potential exposure of approximately 0.416 mg HCN. For a 70‐kg adult, this equates to about 5.94 *μ*g HCN/kg body weight, which is below the acute reference dose for cyanide established by the European Food Safety Authority (EFSA) of 20 *μ*g/kg body weight [47]. It should be emphasised that this estimate represents a deliberately conservative upper‐bound scenario and is likely to overestimate actual exposure for most sweet cultivars, which typically contain lower amygdalin concentrations. Nevertheless, considering the substantial natural variability of cyanogenic glycoside content among apricot kernels, direct quantification of amygdalin should be included in future studies to enable a more accurate assessment of consumer safety. Until such data become available, moderate consumption is advisable, particularly for children and other potentially sensitive population groups.

## 4. Conclusion

Sweet apricot kernels are a valuable source of unsaturated fatty acids, particularly oleic and linoleic acids, as well as proteins, essential amino acids, dietary fibre, minerals and bioactive compounds such as phenolics and vitamin C. Significant differences among the investigated apricot cultivars were observed in fat, glucose and fructose contents, as well as in fatty acid composition. The Kurmayi cultivar generally exhibited higher concentrations of fat and sugar contents, whereas the Suhany cultivar was characterised by lower fat and sugar content, which may be advantageous for consumers seeking low‐energy food options. Between unsulphured and sulphured Kurmay samples, no significant differences in macronutrient levels were observed. The significant differences were in TPC and ascorbic acid content, which are related to the antioxidant properties of sulphates.

Apricot kernels were characterised by a favourable fatty acid profile dominated by oleic and linoleic acids, confirming their high nutritional value and similarity to other oil‐rich seeds and nuts. Their amino acid profile was well balanced, with glutamic acid, aspartic acid and arginine being identified as the major amino acids in the analysed samples. Essential amino acids account for up to 45.3% of the total amino acid content. Their overall nutrient composition was comparable to that of almonds, although differences in individual amino acid and fatty acid profiles reflected species‐specific characteristics. The kernels also represented a notable source of minerals, particularly Cu, Mg and Zn. Furthermore, the presence of phenolic compounds and ascorbic acid contributed to the antioxidant activity of apricot kernels. Taken together, these findings highlight the potential of sweet apricot kernels as sustainable ingredients for the development of functional foods with added nutritional value. Finally, the compositional data generated in this study provide new information on sweet apricot kernels and may contribute to the updating and improvement of national and international food composition databases. Future studies should include direct quantification of amygdalin to enable a more comprehensive assessment of both nutritional value and consumer safety.

## Author Contributions

**Jamila Smanalieva:** conceptualisation, methodology, investigation, data validation, writing – original draft preparation, writing – review and editing. **Janyl Iskakova:** conceptualisation, methodology, investigation, validation, writing – review and editing. **Nurzat Shaikieva:** methodology, investigation, writing – review and editing. **Anke Foerster:** investigation, data validation, writing – review and editing. **Anne Hellwig:** methodology, data validation, writing – review and editing. **Thomas Henle:** conceptualisation, resources, writing – review and editing.

## Funding

This study was funded by the German Federal Ministry of Research, Technology and Space (FKZ: 01LZ2201B). Open Access funding enabled and organized by Projekt DEAL.

## Conflicts of Interest

The authors declare no conflicts of interest.

## Data Availability

The data that support the findings of this study are available upon request from the corresponding author.
